# Supplementary material for: Splice-Junction-Based Mapping of Alternative Isoforms in the Human Proteome
Source: Cell Rep. Author manuscript; Available in PMC 2020 Jan 15. (PMC6961840; doi:10.1016/j.celrep.2019.11.026)
Supplement: 2 [file NIHMS1546469-supplement-2.zip › DF1/PXD006675/AtrialSeptum/AtrialSeptum_32_TPM1_AISEELDHALNDMTSGP.pdf]

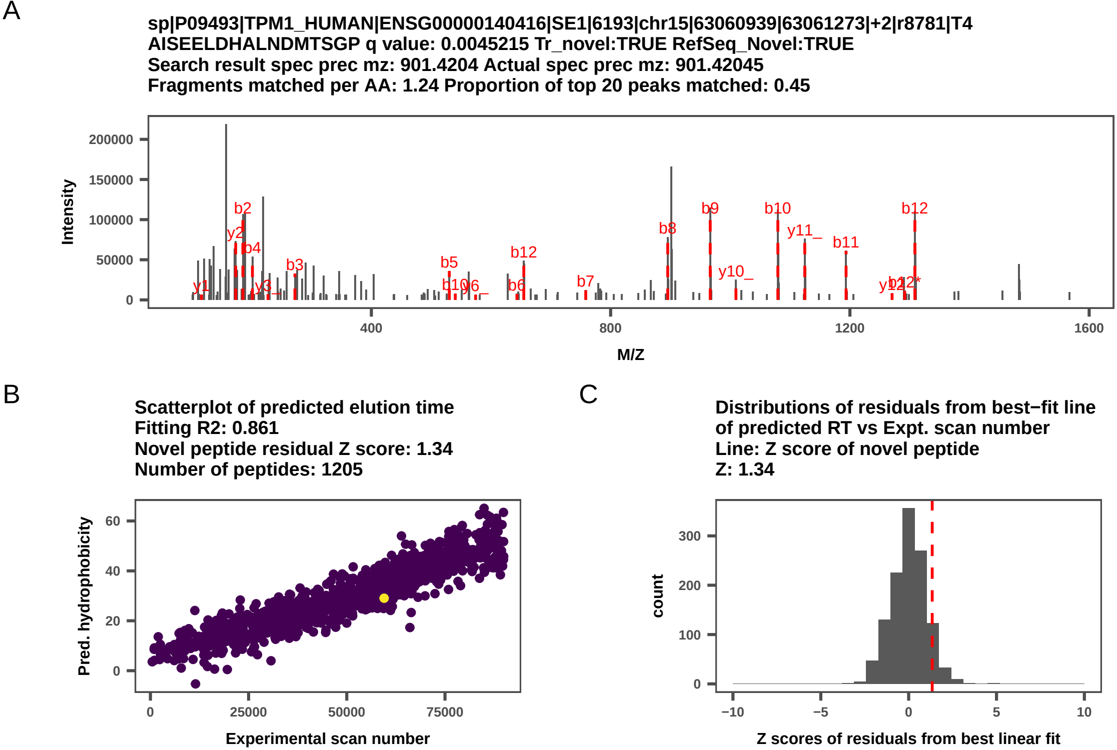
A
sp|P09493|TPM1_HUMAN|ENSG00000140416|SE1|6193|chr15|63060939|63061273|+2|r8781|T4
AISEELDHALNDMTSGP q value: 0.0045215 Tr_novel:TRUE RefSeq_Novel:TRUE
Search result spec prec mz: 901.4204 Actual spec prec mz: 901.42045
Fragments matched per AA: 1.24 Proportion of top 20 peaks matched: 0.45
Intensity
200000
150000
100000
50000
0
M/Z
400
800
1200
1600
b2
y2
b4
y1
y3_
b3
b5
b10
y6_
b12
b6
b7
b8
b9
y10_
b10
y11_
b11
b12
y12
b12*
B
Scatterplot of predicted elution time
Fitting R2: 0.861
Novel peptide residual Z score: 1.34
Number of peptides: 1205
Pred. hydrophobicity
60
40
20
0
0
25000
50000
75000
Experimental scan number
C
Distributions of residuals from best−fit line
of predicted RT vs Expt. scan number
Line: Z score of novel peptide
Z: 1.34
count
300
200
100
0
−10
−5
0
5
10
Z scores of residuals from best linear fit
